# The management of acne vulgaris in young people in primary care: A retrospective cohort study

**DOI:** 10.3389/fmed.2023.1152391

**Published:** 2023-03-20

**Authors:** Aminath Shiwaza Moosa, Shu Fen Lim, Yi Ling Eileen Koh, Wai Keong Aau, Ngiap Chuan Tan

**Affiliations:** ^1^SingHealth Polyclinics, Singapore, Singapore; ^2^SingHealth-Duke NUS Family Medicine Academic Clinical Program, Singapore, Singapore

**Keywords:** primary care (PC), adolescecnt/young adult literature, prescribing pattern, referral to specialist, acne (acne vulgaris), antibiotic monotherapy

## Abstract

**Background:**

Acne vulgaris (acne) is common among young persons (YPs). Clinical practice guidelines are available for acne management to minimize their physical and psychological impact. However, evidence of adherence to these guidelines is sparse in primary care practices. The study aimed to determine the demographic profile of YPs who sought primary care consultations for acne, their related prescriptions and referrals to specialists for further management.

**Method:**

A retrospective study was conducted using data from a cluster of eight public primary care clinics in Singapore. Demographic, clinical, prescription, and referral data were extracted from the electronic health records of YPs aged 10–29 years with a documented diagnosis of acne (ICD-10 classification) from 1st July 2018 to 30th June 2020. The data were reviewed, audited for eligibility criteria, and de-identified before analysis.

**Results:**

Complete data from 2,700 YPs with acne were analyzed. Male (56.1%) YPs and those of Chinese ethnicity (73.8%) had the most frequent attendances for acne. The mean and median age at presentation was 19.2 (standard deviation = 4.3) and 19 (interquartile range = 16–22) years, respectively. Only 69.7% of YPs received an acne-related medication; 33.5% received antibiotic monotherapy; 53.0% were prescribed oral doxycycline, 35.0% acne cream (combination of sulfur, salicylic acid, and resorcinol), and 28.4% benzoyl peroxide 5% gel; 54.3% of those treated with antibiotics were prescribed with a shorter duration than recommended; 51.3% were referred to a dermatologist on their first visit, and 15.8% had more than one visit.

**Conclusion:**

Acne management for YPs can be enhanced with refresher training among primary care physicians for better adherence to its clinical practice guidelines.

## Introduction

Acne vulgaris (henceforth acne) is a common chronic skin disease faced by young persons (YPs). YP is defined as those aged 10–24 years by United Nations, but its definition varies across different localities and populations (1). According to the Global Burden of Disease (GBD) study, approximately 85% of those aged between 12 and 25 years suffer from acne (2). Similarly, in a community-based study in Singapore, acne was reported in 88% of participants aged 13–19 years (3).

Acne is an inflammatory condition with underlying hyperseborrhoea, abnormal follicular keratinisation and Cutibacterium acnes (C. acne) proliferation in the pilosebaceous unit (4). It results in pimples, erythema, scars, and hyperpigmentation in often noticeable areas like the face, neck, and upper torso (5). The resultant psychological distress, especially during the tumultuous adolescent years of developing self-identity can be significant. Acne in YPs is associated with depression, anxiety, and reduced quality of life. (6, 7) Evidence suggests that appropriate acne treatment can alleviate these negative psychological consequences (8).

Treatment of acne ranges from topical to oral medications that require use over a period of time before results are evident. Acne treatment guidelines recommend the judicious use of oral or topical antibiotics and avoidance of monotherapy to reduce the risk of developing antibiotic resistance (3, 9, 10). Should oral antibiotics be warranted, guidelines suggest the use of oral antibiotics for at least 6 weeks for noticeable clinical improvement and not to exceed 3–4 months. Oral antibiotics should be used in combination with non-antibiotic topical agents (9–11).

The outcomes of acne treatment vary with skin type. YPs with darker skin types have the potential for cosmetically disturbing complications, including post-inflammatory hyperpigmentation, scarring, and keloid development (12, 13). These disfiguring cosmetic complications may considerably impact the quality of life (14). Such complications are challenging to treat and are associated with a high cost of comprehensive management (15). The cost of acne scar treatment in China can range from USD 100 to 200 for an individual session, with most patients requiring three to four such sessions (15). In the United Kingdom, acne scar treatment can cost more than GBP 3000 (16). In Singapore, treatment of acne and its associated dermatological complications can be as high as USD 3000 (17). Thus, considering the risk of cosmetic complications and its resultant psychosocial and financial impact, appropriate early therapy is crucial, and under-treatment should be avoided in this vulnerable population to attain higher care satisfaction (18, 19).

Primary care physicians (PCPs) are well placed at the front line of healthcare to identify patients with moderate to severe acne when the latter present for any preventive or acute medical condition. Most mild to moderate acne cases can be treated in primary care. A qualitative study among PCPs in the United States showed that they were comfortable treating mild to moderate acne without needing to refer to a dermatologist (20). Guidelines recommend referring to a specialist in severe or recalcitrant cases not responding to first-line treatments, severe scarring, or psychological or physical distress (9, 11).

In Singapore, PCPs are trained to manage Asian patients across all age groups for common dermatological disorders such as acne and to counsel them on related socio-psychological impact. Undergraduate, postgraduate training and specialized dermatological training are available to equip them with the knowledge and skills to handle patients with acne (21, 22). Nonetheless, information on acne management by local PCPs and their adherence to recommended acne clinical practice guidelines (CPG) are sparse.

It is essential to evaluate the management of acne by primary care physicians and assess alignment of their management practices to the current clinical practice guidelines. Thus, the study aimed to determine the demographic profile of YPs who consulted PCPs for acne, their related prescriptions and referrals to specialists for further management. This will enable us to identify any discrepancies between clinical practice and best available evidence for acne management. In addition, identification of YPs at risk of acne-related complications, especially those with darker skin types among the local multi-ethnic Asian YPs and those with suboptimal treatment, provides opportunity for intervention to enhance their care.

## Methods

### Study design

A retrospective cohort study was conducted to assess the demographic profile, therapeutic management and referral pattern of YPs attending SingHealth Polyclinics (SHP) for acne from 1st July 2018 to 30th June 2020.

### Study sites and population

The study sites comprise eight public primary care clinics (polyclinics) located in the central and eastern regions of the island state. Based on the institution’s electronic medical record (EMR) system and business database, these polyclinics managed 4.2 million patient attendances in 2020, with a quarter million patients in the age group of 10–29 years.

#### Data extraction and processing

In SHP, physicians enter clinical notes and diagnoses, order diagnostic tests, prescribe medications, and document specialist referrals in EMRs using the Sunrise Clinical Manager® electronic platform. Patient sociodemographic characteristics are captured separately in the Outpatient Administrative System (OAS). These clinical and administrative data from multiple healthcare transactional systems are transferred into a single enterprise data repository known as the Electronic Health Intelligence System® (eHINTS).

For this study, the records of YP aged 10–29 years who presented to SHP from 1st July 2018 to 30th June 2020 were extracted from the eHINTS database. The data extracted included information on YPs’ demographics, acne diagnosis, acne-related medications (ARM), duration of prescribed ARM, frequency of follow-up visits, and referrals to specialist care for acne. These data were extracted by the SHP Research Informatics team using the Extract, Transform, and Load (ETL) database function of the eHINTS. The data collated were de-identified and audited for eligibility criteria by trusted third parties before data merging and analysis.

### Outcome definition

Outcomes measured were demographics of YPs, number of cases diagnosed with acne according to ICD-10 classification, prescribing patterns of ARM, frequency of follow-up visits during the study period and proportion of referrals to specialist care such as dermatology and psychiatry. The demographics of YPs comprised nationality, age, gender, and race. Prescribing patterns of ARM included the proportion of ARM prescribed, ARM prescribed as monotherapy, ARM prescribed at the first visit and duration of oral antibiotics.

### Data analysis

Descriptive statistical analysis was used to describe the YP demographics, prescribing patterns of ARM, frequency of follow-up visits, and proportion of referrals to specialist care. Chi-square test was used to compare the demographics of the acne study population with the general patients in SHP. A value of *p* of less than 0.05 is considered statistically significant. All analyses were performed using IBM SPSS version 27.0.

## Results

### Demographic information of young persons aged 10–29 years

Table 1 details the demographic profiles of the YPs who were managed at the study sites during the study period. Of the 103,437 YPs aged 10–29 years who attended SHP during the study period, 2,700 (0.03%) were diagnosed with acne. YPs with acne were mostly those aged 10–19 years (54%), males (56.1%) and Chinese ethnicity (73.8%). The mean age at presentation was 19.2 years, with a standard deviation of 4.3 years. The median age at presentation was 19 years, with an interquartile range of 16–22 years.

**Table 1:** Demographic information of young persons aged 10–29 years seen in SHP during the study period.

Demographic	Demographic characteristics of YP (%)			YPs with acne	All YPs in SHP	Value of *p*
Total	2,700 (100)	103,437 (100)	
Nationality			<0.001
Singapore	2,615 (96.9)	97,446 (94.2)	
Non-Singaporean	85 (3.1)	5,991 (5.8)	
Age (Years)			<0.001
10–19	1,459 (54.0)	40,859 (39.5)	
20–29	1,241 (46.0)	62,578 (60.5)	
Gender			0.001
Male	1,516 (56.1)	54,723 (52.9)	
Female	1,184 (43.9)	48,714 (47.1)	
Race			<0.001
Chinese	1992 (73.8)	61,802 (59.7)	
Malay	413 (15.3)	25,306 (24.5)	
Indian	174 (6.4)	9,227 (8.9)	
Other	121 (4.5)	7,102 (6.9)	

Table 2 lists the consultations for acne by gender and age. Males aged 15–19 years made up the highest proportion (24%) of patients with acne and the most frequent attendances (26%) for acne. Males aged 10 to 24 years had more consults than females of the same age group (8–26 vs. 6–15%), while females attended more frequently than males in the older age group of 25–29 years (7 vs. 6%). Males youths in general require more consultations compared to females youths (Male ranging from 1.21 to 1.30, compared to female ranging from 1.09 to 1.22).

**Table 2:** Consultations for acne by age and gender.

Age (in years) and gender	Number of patients (%)	Total consultations (%)	Total consults/Number of patients
10–14 years Female	195 (7%)	213 (6%)	1.09
10–14 years Male	206 (8%)	250 (8%)	1.21
15–19 years Female	393 (15%)	480 (15%)	1.22
15–19 years Male	660 (24%)	856 (26%)	1.30
20–24 years Female	400 (15%)	467(14%)	1.17
20–24 years Male	485 (18%)	611 (18%)	1.26
25–29 years Female	196 (7%)	223 (7%)	1.14
25–29 years Male	165 (6%)	210 (6%)	1.27

### Prescribing patterns

Table 3 shows only 69.7% of YPs received an ARM during their acne visit. The three most frequently prescribed ARMs were doxycycline (53.0%), acne cream (combination of sulfur, salicylic acid, and resorcinol; 35.0%) and benzoyl peroxide 5% gel (28.4%). Of the 1,223 YPs prescribed oral doxycycline, one in three received it as monotherapy. More than half (54.1%) of the YPs were prescribed oral antibiotics, and 33.5% received antibiotic monotherapy. Only one in three (32.4%) YPs was prescribed combined oral antibiotics with topical non-antibiotics.

**Table 3:** Prescribing patterns of acne-related medications.

Acne-related medication	Prescribing at consultation	Prescribed ARM at consultation	Prescribed as single ARM
	*N* (%)	*N* (%)	*N* (%)
Total acne-related medication	2,306 (69.7)	2,306 (100.0)	1,389 (60.2)
No acne-related medication	1,004 (30.3)	-	-
Topical antibiotics			
Clindamycin gel	4 (0.1)	4 (0.2)	1 (0.1)
			
Total Topical non-antibiotics	1805 (54.5)	1805 (78.3)	922 (66.4)
Acne Cream^1^	806 (24.4)	806 (35)	400 (28.8)
Benzoyl Peroxide 2.5%	532 (16.1)	532 (23.1)	254 (18.3)
Benzoyl Peroxide 5%	654 (19.8)	654 (28.4)	268 (19.3)
Adapalene 0.1%	11 (0.3)	11 (0.5)	1 (0.1)
			
Total Oral antibiotics	1,247 (37.7)	1,247 (54.1)	466 (33.5)
Doxycycline	1,223 (36.9)	1,223 (53)	455 (32.8)
Erythromycin	23 (0.7)	23 (1)	11 (0.8)
Tetracycline	3 (0.1)	3 (0.1)	0
			
Oral antibiotic + topical non-antibiotic	747 (22.6)	747 (32.4)	-
Oral antibiotic + topical antibiotic	2 (0.1)	2 (0.1)	-
Topical antibiotic + topical non-antibiotic	4 (0.1)	4 (0.2)	1 (0.1)

1 Acne cream = cream with a combination of sulfur, salicylic acid, and resorcinol.

Prescribing at consultation = total specific ARM* (or no ARM) prescribed visits/ total patient visits for acne.

Prescribed ARM * at consultation = Visits with specific ARM* prescribed/ All visits with ARM* prescribed.

Prescribed as single ARM*= Visits with only one ARM* prescribed

* ARM, acne-related medication.

**Figure 1:**
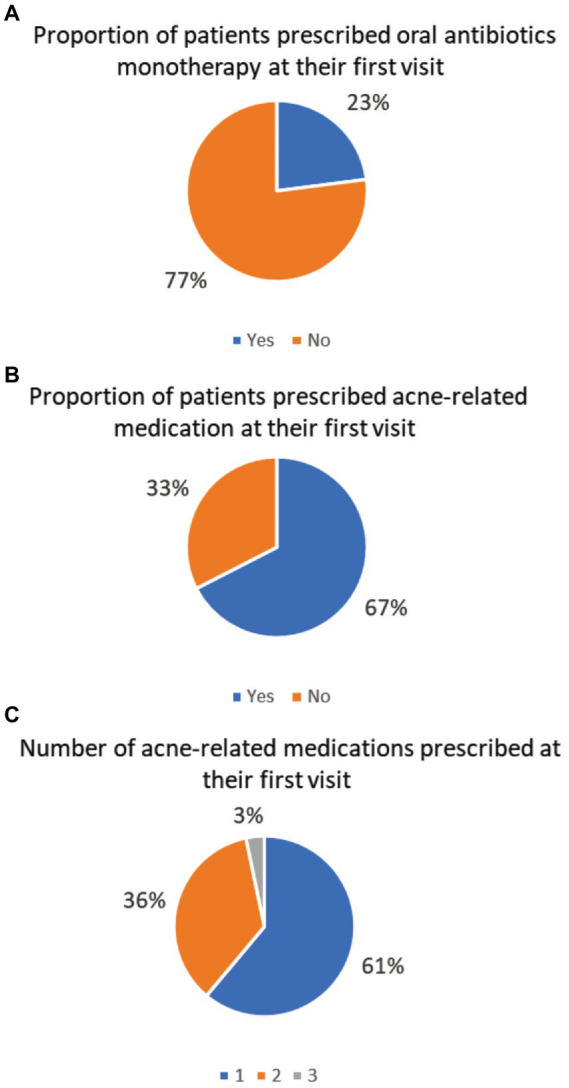
**(A-C)** Prescribing patterns of acne-related medications on patients’ first visit.

The Figure 1 illustrates the prescribing patterns of ARM on YP’s first visit. On their first visit, 67 and 61% of the YPs received any ARM (Figure 1B) and only one ARM (Figure 1C) on their first visit, respectively. Almost one in four (23%) YPs received oral antibiotics as a single medication at their first visit (Figure 1A).

59.8% of the YPs received an inappropriate antibiotic duration (less than 6 weeks or more than 16 weeks). Among these YPs, 54.3% received antibiotics for an insufficient period of less than 6 weeks, and 5.5% received them for more than 16 weeks.

### Referral to specialist care and frequency of follow-up

More than half of the YPs (56.9%) were referred to specialist care on their first visit. Among them, 96.9% of referrals were to a dermatologist. Almost half (48%) of the latter did not receive a trial of therapy. Only 15.8% of the YPs had more than one follow-up visit. The details on the referral and frequency of follow-up are provided in Table 4.

**Table 4:** Follow-up of patients with acne diagnosis and referral to specialist care.

	Number of patients, *N* (%)
Frequency of follow-up visits in SHP	
One visit	2,274 (84.2)
Two or more visits	426 (15.8)
	
Total referred to a specialist	1,535 (56.9)
Referral to specialist done at the first visit	1,385 (51.3)
Referral to specialist done at subsequent visits	178 (6.6)
Referred to dermatologist	1,487 (96.9)
Referral to psychiatrist	2 (0)

## Discussion

Male (56.1%) YPs and those of Chinese ethnicity (73.8%) had the most frequent attendances for acne. A study by Tan et al. represents a similar ethnic distribution among patients with acne in Singapore (3). However, it may not be an accurate indication of an increased prevalence of acne among the Chinese population. The ethnic distribution mirrors the general demographics of Singapore, where 78% of the population is Chinese, 14% Malay, 9% Indian, and 3% other races (23). Similar to the present study, earlier studies report a higher prevalence of acne in younger males than females (24–26). In contrast, women older than 20 were more likely to report acne than men (24, 27).

In younger males, comedonal acne is more frequent and severe compared to inflammatory disease in older females (28–30). These findings are consistent with the natural history of acne. It was reported that acne prevalence and severity correlated with pubertal maturation in young adolescent males (29). Acne is more severe in males than in females in the late teens, as androgen is a potent stimulus to sebum production and severe acne (31). Thus, male YPs tend to present for medical care at younger age compared to females. In addition, severe acne is associated with increased depression, anxiety, poor self-image, and poor self-esteem in this age group (6). Psychiatric symptoms are more common in severe acne and the later stages of puberty (32). Awareness of the presence of acne in this young male population may provide a rationale for early intervention with optimum treatment and prevention of its associated psychosocial burden.

Almost a third of the YPs did not receive acne-related medications, while one in three received oral antibiotics as monotherapy. Nearly half of the YPs received shorter antibiotics courses than recommended. The prescribing patterns did not meet the recommended CPG to achieve optimum management of acne in this study population. Local CPG recommends a combination of oral antibiotics and topical non-antibiotics for moderate acne treatment. The CPG also recommend doxycycline be used as a first-line oral antibiotic (9, 11). Consistent with the latter, doxycycline was the most frequently prescribed oral antibiotic in the current study. Previous cohort studies also revealed that antibiotic was commonly prescribed alone without combined topical treatment at the initial acne consultation (33, 34). Duncan et al. alluded to uncertainties among PCPs toward the effectiveness, acceptability of topical treatments, and their associated side effects (35). Nonetheless, a Saudi Arabian study reported that PCPs had poor knowledge and practices in managing acne vulgaris (36). The prescribing behavior of local PCPs in this study reflects their competency in acne treatment of YPs.

Alternatives to antibiotics are available for acne treatment, such as topical Tretinoin, but such medication is not in the polyclinic pharmacy formulary. The PCPs can offer a prescription for patients to purchase at any pharmacy. Aside from an awareness of the wide availability and affordability of topical acne-related medication off the shelves from pharmacies without a prescription, PCPs generally regard oral antibiotics as the second line of acne treatment. The probability of failed topical treatment before the patient seeks medical consultation is high. PCPs may perceive futility of repeating topical acne treatment. An earlier study among patients in this age group indicated a perception of low effectiveness of topical treatments compared to oral antibiotics and was unaware of antibiotic resistance (37). Perceived pressure from patient is strongly associated with antibiotic prescribing among PCPs (38, 39). While details of interactions were not extracted from the EMR, patients’ desire for oral antibiotics may inevitably influence their prescription among the PCPs.

While PCPs generally are familiar with the choice of oral antibiotics, the results suggest that they seem less aware of its appropriate treatment duration. Inadequate antibiotic duration results in subinhibitory and subtherapeutic antibiotic concentrations, which promote the development of antibiotic resistance and frequent relapse (40, 41). On the other hand, the insufficient antibiotic duration may also reflect a lack of follow-up by patients. Guidelines suggest that patients should be reviewed after 6–8 weeks to assess treatment response and provide support (9, 10). Thus, PCPs may prescribe a trial of medication for a shorter period to determine the patient’s tolerability and treatment response, particularly when incidences of side effects are high. Doxycycline has common adverse effects such as photosensitivity or gastric disturbance. Furthermore, patients can have multiple providers in the local primary healthcare system. Any episodic care in such setting can potentially hinder patients from completing their required period of antibiotic.

Only a minority (15.8%) of YPs had more than one visit for acne, suggesting that longitudinal care is suboptimum in primary care. In contrast, similar cohort studies assessing acne management among primary care practices in the United Kingdom reported a higher follow-up rate; Francis et al. reported a follow-up of one-third of their patients attending for additional acne consultations after their initial visit (33) and Purdy et al. described 21% of young persons with acne following up for a second visit (34). Some patients in the current study may have had follow-up with other physicians or self-medicated with over-the-counter medications. Other patients may have their symptoms resolved with the initial treatment and did not consider a need for follow-up. Young persons often perceive acne as a short-term, self-limiting condition of adolescence, which influences their health and help-seeking behavior for acne (37, 42). Effective communication and shared decision-making to improve patient engagement can improve adherence to medication regimes and promote continuity of care among young persons (43). Furthermore, some patients may have had mild acne that required only topical medications and no follow-up. As acne severity was not assessed in the present study, it is difficult to ascertain if the patients required a long-term follow-up. However, two-thirds of the patients were prescribed an acne-related medication, and more than three-fourths were prescribed an oral antibiotic on their first visit. Thus, the number of patients with mild acne who did not require a follow-up may have been small. A lack of follow-up in primary care could also have been because of the high referral to specialist care after their initial visit.

In a retrospective British cohort study reported that PCPs referred only 8.5% of young patients with acne to a dermatologist. (34) A qualitative study among American PCPs revealed low perceived needs to refer patients for acne management (35). They would treat most patients with mild to moderate acne. According to Charles et al., PCPs can and should handle most patients with acne without a referral (20). In contrast, over half of YPs in the current study were referred to specialists for further treatment despite being the first consultation for acne. This may be attributed to patient, provider or healthcare environment factors. Patients may be presenting with acne that is more severe physically as well as in its’ effects on quality of life and warrant a dermatologist referral. As mentioned previously, Asian patients may have darker skin types that have increased tendencies for acne complications. However, it may also reflect patient preferences for their acne to be managed by a specialist over a primary care provider (38). The significant government subsidizes provided to patients, combined with the ease of obtaining a dermatologist referral and appointment may also influence a patient’s decision in this aspect. In addition, PCPs tend to refer to tertiary care when evidence-based treatment is lacking in primary care setting (20). Despite oral isotretinoin being a medication that can be safely initiated and monitored in primary care, it is currently unavailable at many primary care clinics (44). This could be a reason why PCPs refer to specialist care for such treatment.

Despite the well-recognized psychosocial impact of acne and associated mood disorders, only two patients were referred to a psychiatrist in the present study. These findings may reflect the stigma associated with mental disorder among local Asian adults. Many view mental illness as a personal weakness compared to a medical condition. YPs are adverse to possible social and career repercussions if they seek medical attention to such disorders (45). Nonetheless, patients comfortable with their PCPs may be more willing to divulge their psychological concerns. In addition, a systematic review indicated that people perceived “trivialization” of their acne by healthcare providers, which affected their health-seeking behavior (42). With these barriers, it may not be easy for YPs to approach PCP for psychiatric help. PCPs should pay attention to acne-associated psychosocial and mood disorders and manage the affected YPs sensitively (7).

In addition, the study was based on data collected from a public primary care service institute in Singapore and may not reflect the practice of the primary care physicians in the private sector. Public primary care services in Singapore are provided by government subsidized polyclinics; the services are affordable, with patients paying lower consultation fees and receiving government subsidies for medication and treatments. Certain medications (e.g., oral isotretinoin or dermocosmetics) are not subsidized and not available in the polyclinic drug formulary. On the other hand, private primary care services in Singapore are typically more expensive, with patients paying higher consultation fees and the full cost of medication and treatments. Thus, a more comprehensive range of medications, including oral isotretinoin and dermocosmetics, are available to the patients. These private practitioners may run esthetic clinics, where acne scar treatments such as laser and chemical peels are available. In addition, patients can choose their preferred doctor and have greater flexibility in scheduling appointments. Thus, these differences in availability of services, convenience and healthcare needs may affect patients’ choosing private over public primary care services for acne treatment.

## Strengths and limitations

The strength of this study emerges as among the few real-world report of acne management among Asian YPs in urban primary care practices, leveraging on a large database. The search strategy used an established diagnosis code for searching and data extraction.

However, the reliance on this data depends on the accuracy of the diagnosis recording. In addition, some of the ARM may be used for other conditions (e.g., doxycycline can be used for other skin infections). YPs prescribed these medications were only included if there was a linked diagnosis of acne for the same visit. The use of topical medications or adjuvant therapy could be under-reported as over-the-counter topicals (e.g., Benzoyl peroxide formulations and dermocosmetics) that patients may have been using were unaccounted for. Also, the data on certain medications that were unavailable in the drug formulary such as oral isotretinoin were not captured. In addition, potential drug allergies may prevent certain antibiotics being prescribed. However, the data on drug allegies cannot be extracted from the EMR. Nevertheless, in the presence of drug allergy alternative first-line antibiotic options are available to the physician to treat acne. The study did not assess the severity of the acne. Thus, it was challenging to determine the appropriateness of the referral to specialist, the therapeutic choice and the clinical outcome.

## Conclusion

Acne is common among YPs. More young males presented with acne at public primary care clinics. Oral antibiotics as a single therapy and its short duration were common therapeutic option which was not aligned with CPG. Concurrent use of non-antibiotic topicals was sparse, increasing the risk of antibiotic resistance. Follow-up reviews were few, and high referral rates to specialist care could affect care continuity for YPs with acne. Medical training and refresher course for acne CPG will empower PCPs to optimize acne treatment for YPs.

## Data availability statement

The original contributions presented in the study are included in the article/Supplementary material, further inquiries can be directed to the corresponding author.

## Ethics statement

The studies involving human participants were reviewed and approved by SingHealth Centralized Institutional Review Board (2021/2003). Written informed consent from the participants’ legal guardian/next of kin was not required to participate in this study in accordance with the national legislation and the institutional requirements.

## Author contributions

WA provided the data. YK analyzed the data. AM and SL wrote the first draft of the manuscript. NT actively contributed to revise the manuscript. All authors contributed to the article and approved the submitted version.

## Funding

The study was funded by Seed Grant by the SingHealth Family Medicine Academic Clinical Program. The publication of this study is funded by SingHealth Polyclinic Research Department.

## Conflict of interest

The authors declare that the research was conducted in the absence of any commercial or financial relationships that could be construed as a potential conflict of interest.

## Publisher’s note

All claims expressed in this article are solely those of the authors and do not necessarily represent those of their affiliated organizations, or those of the publisher, the editors and the reviewers. Any product that may be evaluated in this article, or claim that may be made by its manufacturer, is not guaranteed or endorsed by the publisher.

## References

[1] UNFPA (2022). Adolescent and youth demographics:a brief overview. Available at: https://www.unfpa.org/resources/adolescent-and-youth-demographicsa-brief-overview (Accessed November 15, 2022).

[2] Matsubayashi K. Global burden of disease. Lancet. (1997) 350:144–5. doi: 10.1016/s0140-6736(05)61851-x 9228985

[3] Tan HH Tan AWH Barkham T Yan XY Zhu M. Community-based study of acne vulgaris in adolescents in Singapore. Br J Dermatol. (2007) 157:547–51. doi: 10.1111/J.1365-2133.2007.08087.X, PMID: 17655737

[4] Dréno B. What is new in the pathophysiology of acne, an overview. J Eur Acad Dermatol Venereol. (2017) 31:8–12. doi: 10.1111/JDV.14374, PMID: 28805938

[5] Mahto A. Acne vulgaris. Medicine (Baltimore). (2017) 45:386–9. doi: 10.1016/J.MPMED.2017.03.003

[6] Kilkenny M Stathakis V Hibbert ME Patton G Caust J Bowes G. Acne in Victorian adolescents: associations with age, gender, puberty and psychiatric symptoms. J Paediatr Child Health. (1997) 33:430–3. doi: 10.1111/J.1440-1754.1997.TB01635.X, PMID: 9401889

[7] Aktan S md Ozmen E md S B cedil. Anxiety, depression, and nature of acne vulgaris in adolescents. Int J Dermatol. (2000) 39:354–7. doi: 10.1046/J.1365-4362.2000.00907.X, PMID: 10849125

[8] Gieler U Gieler T Kupfer JP. Acne and quality of life—impact and management. J Eur Acad Dermatol Venereol. (2015) 29:12–4. doi: 10.1111/JDV.13191 26059729

[9] Oon HH Wong SN Wee DCAW Cheong WK Goh CL Tan HH. Acne management guidelines by the dermatological society of Singapore. J Clin Aesthet Dermatol. (2019) 12:34–50. PMID: 31531161 PMC6715335

[10] Zaenglein AL Pathy AL Schlosser BJ Alikhan A Baldwin HE Berson DS. Guidelines of care for the management of acne vulgaris. J Am Acad Dermatol. (2016) 74:945–973.e33. doi: 10.1016/j.jaad.2015.12.037 26897386

[11] Moosa AS Quah JHM How CH. Primary care approach to managing acne. Singap Med J. (2021) 62:568–73. doi: 10.11622/smedj.2021225, PMID: 35001135 PMC8804415

[12] Perkins AC Cheng CE Hillebrand GG Miyamoto K Kimball AB. Comparison of the epidemiology of acne vulgaris among Caucasian, Asian, continental Indian and African American women. J Eur Acad Dermatology Venereol. (2011) 25:1054–60. doi: 10.1111/j.1468-3083.2010.03919.x, PMID: 21108671

[13] Davis EC Callender VD. A review of acne in ethnic skin: pathogenesis, clinical manifestations, and management strategies. J Clin Aesthet Dermatol. (2010) 3:24–38. 20725545 PMC2921746

[14] Yazici K Baz K Yazici AE Köktürk A Tot S Demirseren D. Disease-specific quality of life is associated with anxiety and depression in patients with acne. J Eur Acad Dermatol Venereol. (2004) 18:435–9. doi: 10.1111/J.1468-3083.2004.00946.X, PMID: 15196157

[15] Xiao Y Chen L Jing D Deng Y Chen X Su J. Willingness-to-pay and benefit-cost analysis of chemical peels for acne treatment in China. Patient Prefer Adher. (2019) 13:363–70. doi: 10.2147/PPA.S194615, PMID: 30863024 PMC6391120

[16] NGA (UK) Management of acne vulgaris-associated scarring. (2021) Available at: https://www.ncbi.nlm.nih.gov/books/NBK573047/ (Accessed January 14, 2023).

[17] Acne Treatment Singapore (2023). 7 Skin Clinics & How Much They Cost. Available at: https://blog.moneysmart.sg/healthcare/acne-treatment-singapore-cost/ (Accessed January 14, 2023).

[18] Taylor SC Cook-Bolden F Rahman Z Strachan D. Acne vulgaris in skin of color. J Am Acad Dermatol. (2002) 46:S98–S106. doi: 10.1067/MJD.2002.120791 11807471

[19] Callender VD. Acne in ethnic skin: special considerations for therapy. Dermatol Ther. (2004) 17:184–95. doi: 10.1111/J.1396-0296.2004.04019.X, PMID: 15113286

[20] Margolis CF Ramundo ML. Acne management. Primary care physician or dermatologist? Postgrad Med. (1987) 82:139–46. doi: 10.1080/00325481.1987.11700081 2960963

[21] Goh LG Ong CP. Education and training in family medicine: Progress and a proposed national vision for 2030. Singap Med J. (2014) 55:117–23. doi: 10.11622/smedj.2014031, PMID: 24664375 PMC4293980

[22] Chew CH Chee YC. Postgraduate medical education and specialist training in Singapore. Ann Acad Med Singap. (2005) 34:182–9. 16010405

[23] Population and Population Structure (2023) Statistics Singapore. Department of Statistics, Singapore. Available at: https://www.singstat.gov.sg/find-data/search-by-theme/population/population-and-population-structure/latest-data (Accessed January 21, 2023).

[24] Thappa DM Adityan B. Profile of acne vulgaris-a hospital-based study from South India. Indian J Dermatol Venereol Leprol. (2009) 75:272–8. doi: 10.4103/0378-6323.51244, PMID: 19439880

[25] Alanazi MS Hammad SM Mohamed AE. Prevalence and psychological impact of acne vulgaris among female secondary school students in Arar city, Saudi Arabia, in 2018. Electron Physician. (2018) 10:7224–9. doi: 10.19082/7224, PMID: 30214705 PMC6122864

[26] Abo El-Fetoh NM Alenezi NG Alshamari NG Alenezi OG. Epidemiology of acne vulgaris in adolescent male students in Arar, Kingdom of Saudi Arabia. J Egypt Public Health Assoc. (2016) 91:144–9. doi: 10.1097/01.EPX.0000492401.39250.62, PMID: 27749646

[27] Collier CN Harper JC Cantrell WC Wang W Foster KW Elewski BE. The prevalence of acne in adults 20 years and older. J Am Acad Dermatol. (2008) 58:56–9. doi: 10.1016/J.JAAD.2007.06.045, PMID: 17945383

[28] Duquia RP de Almeida HL Breunig JA Souzat PRM Göellner CD. Most common patterns of acne in male adolescents: a population-based study. Int J Dermatol. (2013) 52:550–3. doi: 10.1111/J.1365-4632.2011.05333.X, PMID: 23590370

[29] Lucky AW Biro FM Huster GA Morrison JA Elder N. Acne vulgaris in early adolescent boys: correlations with pubertal maturation and age. Arch Dermatol. (1991) 127:210–6. doi: 10.1001/ARCHDERM.1991.01680020078009 1825016

[30] Khunger N Kumar C. A clinico-epidemiological study of adult acne: is it different from adolescent acne? Indian J Dermatol Venereol Leprol. (2012) 78:335–41. doi: 10.4103/0378-6323.95450, PMID: 22565434

[31] Stathakis V Kilkenny M Marks R. Descriptive epidemiology of acne vulgaris in the community. Australas J Dermatol. (1997) 38:115–23. doi: 10.1111/J.1440-0960.1997.TB01126.X, PMID: 9293656

[32] Purvis D Robinson E Merry S Watson P. Acne, anxiety, depression and suicide in teenagers: a cross-sectional survey of New Zealand secondary school students. J Paediatr Child Health. (2006) 42:793–6. doi: 10.1111/J.1440-1754.2006.00979.X, PMID: 17096715

[33] Francis NA Entwistle K Santer M Layton AM Eady EA Butler CC. The management of acne vulgaris in primary care: a cohort study of consulting and prescribing patterns using the clinical practice research datalink. Br J Dermatol. (2017) 176:107–15. doi: 10.1111/BJD.15081, PMID: 27716910

[34] Purdy S Langston J Tait L. Presentation and management of acne in primary care: a retrospective cohort study. Br J Gen Pract. (2003) 53:525–9. PMID: 14694664 PMC1314642

[35] Platt D Muller I Sufraz A Little P Santer M. GPs’ perspectives on acne management in primary care: a qualitative interview study. Br J Gen Pract. (2020) 71:E78–84. doi: 10.3399/BJGP20X713873, PMID: 33257464 PMC7716869

[36] Alotaibi A Baradah R Alzahrani A Saad A Aldawsari. Knowledge and practice of primary healthcare physicians for management of acne vulgaris in Sudair area, Saudi Arabia. J Egypt Public Health Assoc. (2018) 2017-2018:2454–9142.

[37] Ip A Muller I Geraghty AWA McNiven A Little P Santer M. Young people’s perceptions of acne and acne treatments: secondary analysis of qualitative interview data. Br J Dermatol. (2020) 183:349–56. doi: 10.1111/BJD.18684, PMID: 31701523 PMC7496424

[38] Little P Dorward M Warner G Stephens K Senior J Moore M. Importance of patient pressure and perceived pressure and perceived medical need for investigations, referral, and prescribing in primary care: nested observational study. BMJ. (2004) 328:444–6. doi: 10.1136/BMJ.38013.644086.7C 14966079 PMC344266

[39] Stivers T. Managing patient pressure to prescribe antibiotics in the clinic. Paediatr Drugs. (2021) 23:437–43. doi: 10.1007/S40272-021-00466-Y, PMID: 34410633 PMC8375467

[40] Levy-Hara G Amábile-Cuevas CF Gould I Hutchinson J Abbo L Saxynger L. “Ten commandments” for the appropriate use of antibiotics by the practicing physician in an outpatient setting. Front Microbiol. (2011) 2:1–17. doi: 10.3389/FMICB.2011.00230 22164154 PMC3225075

[41] Ventola CL. The antibiotic resistance crisis: part 1: causes and threats. Pharm Ther. (2015) 40:277. PMC4378521 25859123

[42] Ip A Muller I Geraghty AWA Platt D Little P Santer M. Original research: views and experiences of people with acne vulgaris and healthcare professionals about treatments: systematic review and thematic synthesis of qualitative research. BMJ Open. (2021) 11:e041794. doi: 10.1136/BMJOPEN-2020-041794 PMC7853035 33526498

[43] Lee PY Ng CJ. Practising shared decision making in primary care. Malaysian Fam Phys Off J Acad Fam Phys Malaysia. (2021) 16:2–7. doi: 10.51866/CM0001, PMID: 33948136 PMC8088742

[44] Buckley D Yoganathan S. Can oral isotretinoin be safely initiated and monitored in primary care? A case series. Ir J Med Sci. (2017) 186:315–9. doi: 10.1007/S11845-016-1540-5, PMID: 28070816

[45] Ma QH Parisi JM Joo JH Gallo JJ. Singapore young adults’ perception of mental health help-seeking from mental health professionals and peer supporters. Asian J Psychiatr. (2021) 61:102687. doi: 10.1016/J.AJP.2021.102687, PMID: 34004461

